# Mechanical and Electrical Characterization of Entangled Networks of Carbon Nanofibers

**DOI:** 10.3390/ma7064845

**Published:** 2014-06-23

**Authors:** Arash K. Mousavi, Mark A. Atwater, Behnam K. Mousavi, Mohammad Jalalpour, Mahmoud Reda Taha, Zayd C. Leseman

**Affiliations:** 1 Mechanical Engineering Department, University of New Mexico, Albuquerque, NM 87106, USA; E-Mail: mousavi@unm.edu; 2 Applied Engineering, Millersville University, Millersville, PA 17551, USA; E-Mail: mark.atwater@millersville.edu; 3 Electrical & Computer Engineering Department, University of New Mexico, Albuquerque, NM 87106, USA; E-Mail: behnamkm@unm.edu; 4 Civil Engineering Department, University of New Mexico, Albuquerque, NM 87106, USA; E-Mails: mj95@unm.edu (M.J.); mrtaha@unm.edu (M.R.T.)

**Keywords:** carbon, entangled, nanofibers

## Abstract

Entangled networks of carbon nanofibers are characterized both mechanically and electrically. Results for both tensile and compressive loadings of the entangled networks are presented for various densities. Mechanically, the nanofiber ensembles follow the micromechanical model originally proposed by van Wyk nearly 70 years ago. Interpretations are given on the mechanisms occurring during loading and unloading of the carbon nanofiber components.

## 1. Introduction

One-dimensional carbon materials are available in many high-end commercial products [1,2] and continue to be a source of ongoing research [3,4,5]. This is due to their remarkable material properties. The two main instances of this type of material are carbon nanotubes and carbon nanofibers. Carbon micro- and nanofibers are a common component in high-strength, lightweight fiber-reinforced composites [6,7,8,9]. Less studied are the properties of an interwoven assemblage of the nanofibers, which behaves as a coherent, nonwoven component.

Synthesis methods for creating carbon nanotubes [10] and nanofibers [10,11,12] commonly produce random networks of tangled fibers. These entangled fibers do not have fixed connections with adjoining fibers (*i*.*e*., they are not cross-linked). Thus, the entangled fibers comprise a random network similar in nature to a nonwoven mass of fibers found in common textile processes. Random networks of textile fibers are commonly modeled using a micromechanical model originally developed by van Wyk in 1946. In 2009 an effort was undertaken to mechanically test a “tangle” of carbon nanotubes [3]. This effort demonstrated that the van Wyk model [13] matched the stress *vs*. change in fiber volume fraction for the loading portion of the curve. It also showed that the van Wyk model did not match the unloading portion of the curve. Allaoui *et al*. [3] surmise that this is due to the dissipation of adhesive energy as contacts break.

The van Wyk model was proposed in 1946 [13] and incorporates fiber bending at contact points in the random network and the creation of new fiber-to-fiber contacts, but ignores other effects such as friction and fiber sliding. Functionally, van Wyk’s model proportionally relates the stress applied to the random fiber network, σ, to the fiber volume fraction, µ, to the third power:

(1) $$\sigma =k_{p}({µ}^{3}-{µ}_{0}^{3})$$

where *k*_p_ is a constant that contains information on fiber characteristics and µ_0_ is the initial fiber volume fraction before compression. This model describes the behavior of a large entangled network of randomly oriented fibers. This amazingly simplistic model has been found to be applicable to many different types of random fiber networks such as short hollow pulp fiber networks [14] and textile reinforcements for composites manufacturing [15].

The electrical response of a random fiber network to an applied stress is not commonly studied, because most types of fibers in the textile industry are not conductive. Thus, there is a lack of studies and modeling addressing this topic. In this paper, the electrical properties are measured in both tension and compression. Sudden changes in electrical resistance seen in tension tests can be used as a method of evaluating the mechanical situation and integrity of the material. For compression tests, the electrical resistance is shown to approach the conductance of amorphous carbon when the carbon nanofibers are under their highest compressive loads. Additionally, the mechanical response of the entangled networks of carbon nanofibers is studied in tension and compression. A discussion of the tensile response is qualitative while the compressive response is modeled using the van Wyk Model. The compression tests reported here are performed in an open die configuration under high stresses (~10 MPa). During loading, it was verified that the sample kept its integrity even at the highest loads. Furthermore, it is shown that the van Wyk Model Equation (1) for a random entanglement of fibers is capable of describing the response of the material with considerable accuracy.

## 2. Experimental Section

### 2.1. Carbon Nanofiber Synthesis

Bulk, nonwoven components comprised of carbon nanofibers were synthesized using a method previously developed by Atwater *et al*. [10]. Briefly, Pd nanoparticles are dispersed in a rectangular steel mold and then heated in a furnace in an inert environment. Once heated to 550 °C, a mixture of ethylene and oxygen is flown over the catalyst. By varying the Pd particle loading in the mold and the time of gas flow differing densities of carbon nanofiber entanglements were synthesized. Sample densities were determined geometrically by weighing samples of a known volume.

**Figure 1:**
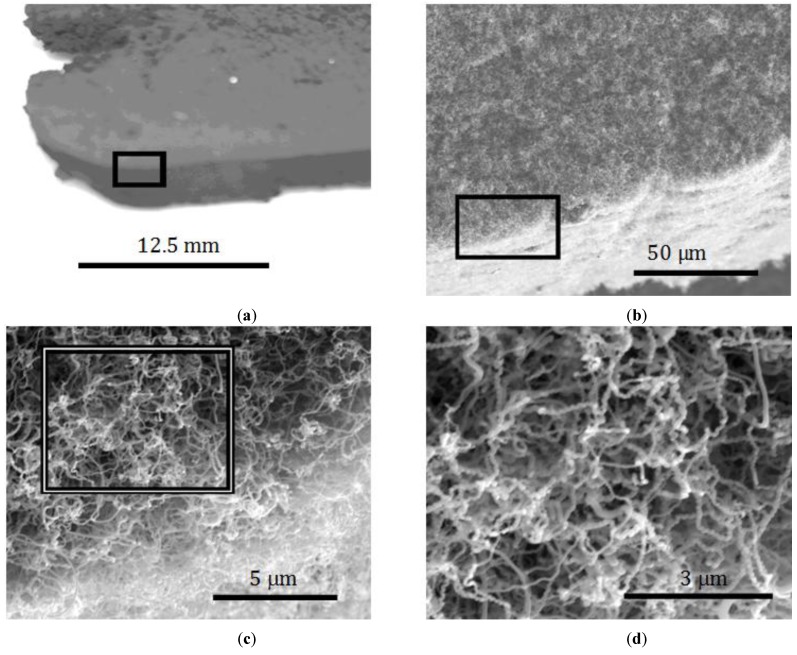
Carbon Nanofiber Network at increasing magnification, boxes indicate section in next image (**a**) 1×; (**b**) 1000×; (**c**) 10,000×; (**d**) 20,000×.

An example of the as-grown samples is shown in Figure 1. Here it can be seen that a relatively large (many centimeters) component can be easily attained with this process. When examined using SEM, it can be seen that the larger structure is actually comprised of much smaller (*ca*. 100 nm diameter), carbon nanofibers, e.g., Figure 1d. Additional characterization of the fibers’ properties is contained in References [11,12]. The process, then, creates a multi-scale material with the ability to control both nanoscale and macroscale features.

### 2.2. Mechanical and Electrical Characterization Setups

Mechanical and electrical characterization was conducted simultaneously for samples of differing densities. In order to accomplish this, a custom setup was required for both the tensile and compressive tests. Tensile samples were tested under relatively low loads, <45 N, while compression tests were to a load as high as 200 N.

Tensile tests were conducted on an Instron 1101 with a 45 N load cell using a constant displacement of 1.27 mm/min. Sample densities tested were 0.125 and 0.131 g/cc. Carbon nanofiber samples with a cross sectional area of 5 mm × 25 mm were clamped between two electrically isolated grips at a distance of 20 mm apart. A constant current of 250 mA was applied to the samples and the voltage was recorded in order to determine the resistance of the sample as a function of load. 

Compression tests were performed on a larger capacity Instron with a 44.5 kN load cell with a constant crosshead displacement of 0.252 mm/min. The 0.40 g/cc cylindrical sample was tested in an open die configuration with a diameter of 4.7 mm and thickness of 5 mm. Electrical measurements were made in the same manner as for the tensile experiments.

## 3. Mechanical Properties

### 3.1. Tensile Tests

**Figure 2:**
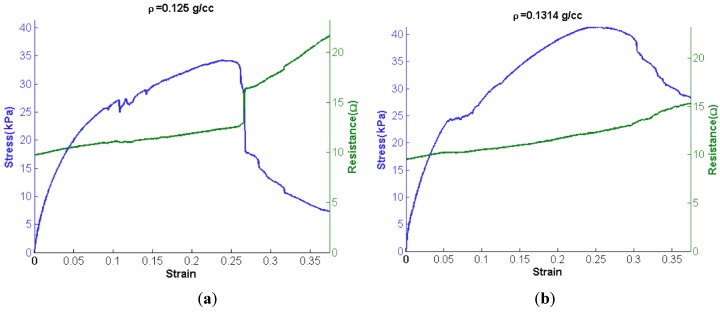
Tensile behavior of carbon nanofiber network under tension, accompanied by resistance measurements as a function of strain (mm/mm). (**a**) Low density network; (**b**) High density network.

Tensile loading curves for two different densities of nanofibrous nonwoven carbon are shown in Figure 2. The stress-strain curves are accompanied by their corresponding resistance measurements. Judging from SEM characterization (e.g., see Figure 1), no fiber is expected to span the gage length of the sample, and therefore no fibers are considered rigidly held by both grips. On that assumption, loading of the samples must be a result of friction between the fibers and mechanical interlocking.

Tensile loading of the samples diplays several interesting behaviors. For both samples, a smooth monotonic increase is seen during loading initially. This smooth portion of the loading curves is then followed by data that is generally increasing, but contains several discontinuities. These discontinuities are attributed to avalanches of fiber pullouts. After a critical number of fibers have pulled out, a maximum load is reached, and any increase in displacement results in nothing but decreases in load.

In order to more succinctly compare the behavior of these two samples five material properties are examined: (1) tangent modulus; (2) maximum stress; (3) maximum strain; (4) failure stress and (5) failure strain. Failure is defined as a decrease in the load sustained by the sample. Table 1 compares the compressive moduli and tangent moduli attained from tensile tests. Though the two samples have nearly identical densities and remarkably similar material properties, as listed in Table 1, there is a marked difference in the failure of the two samples. The 0.125 g/cc sample has a massive avalanche of pulled out fibers as is evidenced by a sudden drop in load after reaching σ_u_; this is also accompanied by a large jump in resistance of the sample. The 0.131 g/cc sample appears to have a fairly gradual pullout of fibers as it continues to fail after having reached its σ_u_.

**Table 1:** Mechanical Properties of entangled network of carbon nanofibers.

ρ (g/cm^3^)	*E(*kPa*)*	*σ_v_ (*kPa*)*	σ_u_ (kPa)	Linear range Percentage (% of σ_u_)
0.125	600	13.4	34.2	39.2%
0.131	627	16.9	41.3	40.8%

The resistance of the entangled network of the carbon nanofibers increases almost linearly with increasing tensile load until there is a sudden drop in the load (stress). This is a clear indication that fibers are pulling out from one another. This reduces the number of fibers in contact as well as the number of contacts per fiber. Once a threshold limit is met, then many fibers separate from one another simultaneously—an avalanche of fiber pullouts. Again, this is most noticeable for the 0.125 g/cc sample near its σ_u_. 

### 3.2. Compression Tests

**Figure 3:**
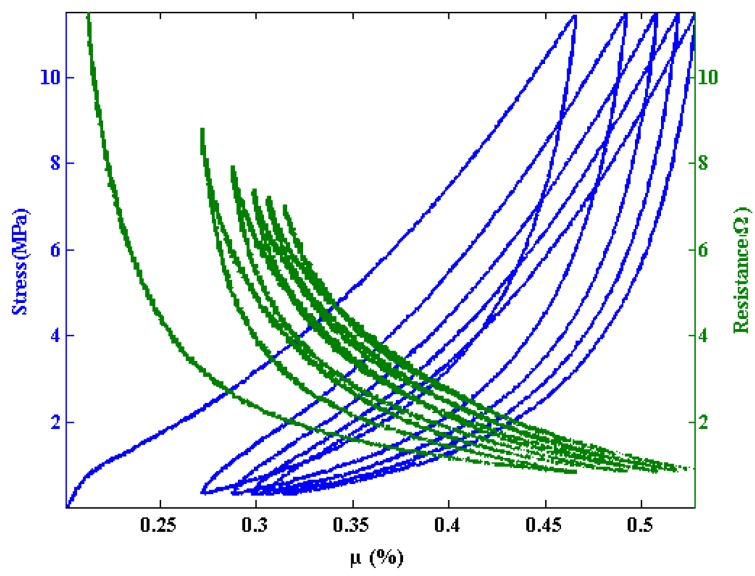
Five compression cycles (continuous loading and unloading) of a 0.40 g/cc carbon nanofiber tangle sample. Data is displayed as pressure *vs*. percent fiber volume fraction (µ).

**Figure 4:**
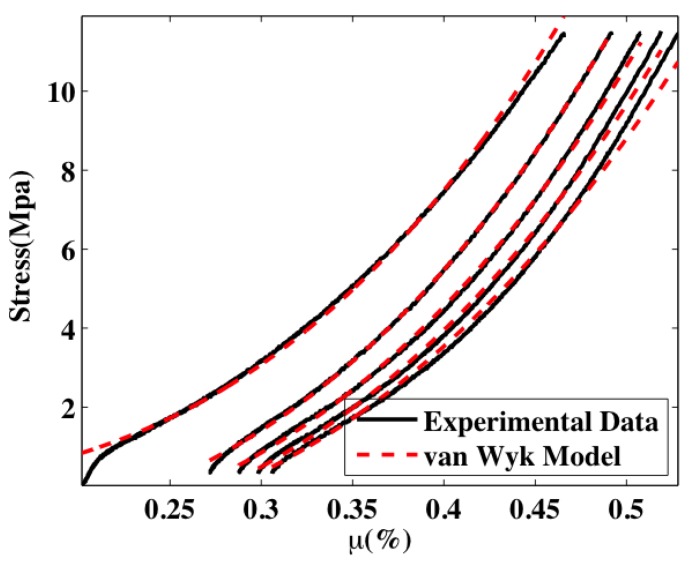
Loading portions of the curves in Figure 3 with fits to the van Wyk model.

Five compressive loading cycles are shown for a sample with a of density 0.40 g/cc in Figure 3. Cycle 6 and subsequent cycles display the similar hysteresis between the loading and unloading portion of each cycle, but nearly fall on top of one another. The stress *versus* percent fiber volume fraction (µ%) are accompanied by their corresponding resistance measurements. The resistance *vs*. µ% curves follow similar trends, as seen by Atwater *et al*. [10]. Additionally, the loading portions of the curves in Figure 3 are fit with the van Wyk model, Equation (1). The agreement between the experimental data and model demonstrate the utility of the model and also show that the mechanisms modeled in the van Wyk interpretation are responsible for the defromation behavior of the entanglement of the carbon nanofibers.

van Wyk’s model describes the bending and creation of new contacts in a random network of fibers. This is appropriate for the compressive loading of the fibers as evidenced by the fit of van Wyk’s model to the data in Figure 4. Unloading of the fiber shows a considerable amount of hysteresis and is not modeled well by van Wyk’s model (and is not displayed). For unloading, it is likely that adhesion and friction are the main contributors to this hysteresis, as is the case with carbon nanotubes [3].

Even within the loading event, it appears that bending and the creation of contacts contribute different amounts to the loading history of the sample as the fiber volume fraction increases. For lower values of µ (and load) the samples are more porous, *i*.*e*., there are more air gaps between the carbon nanofibers. Thus, as the sample is compressed, the fibers bend into the empty spaces more readily—bending is more dominant at lower values of load and µ. Thus, the model does not work as well for lower values of load and µ. As the compression continues, new contacts are made at an increasing rate. In fact, it has been recently shown that the rate of increase of the number of contacts is linearly proportional to µ [16]. Thus for higher values of load and µ the van Wyk model is more applicable and therefore more accurate. A considerable amount of hysteresis exists between the loading and unloading cycles in Figure 3. Adhesion (friction) is attributed to this hysteresis. Adhesive forces are commonly known to cause sticking between individual micro and nanodevices, commonly referred to as ‘stiction failure’ in the literature [17,18,19]. Because of the small scale of the fibers (*i*.*e*., *ca*. 100 nm diameter), van der Waals forces, hydrogen bonding, *etc*. may have an appreciable adhesive effect. Allaoui *et al*. [3] also consider this to be the source of adhesion between fibers and the culprit for hysteresis in carbon nanotube tangles.

Adhesion between fibers leads to hysteresis while the bending of individual fibers is the main source of elastic energy storage. As the load increases more fibers are being influenced to move, but their motion is impeded due to restrictions from adjacent fibers. For the highest loads, energy is dissipated by irreversible friction and sliding [20]. However, when unloading occurs, only the elastically stored energy can be recovered. Thus a small reverse movement in the compression platen results in a large drop in load as seen in all unloading curves. For lower values of µ (strain) the elastic energy in the fibers becomes considerable and pushes back on the compression platen and a large movement of the platen results in a smaller change in load.

The resistance of the entangled network decreases nonlinearly as the compressive load is applied in Figure 3. An asymptotic value is approached for the highest loads. Considering the sample size, the resistivity of the sample approaches 2.7 × 10^−3^ Ω·m, which is approximately three to five times that of amorphous carbon (5 × 10^−4^–8 × 10^−4^ Ω·m) [21,22]. Physically, the sample is being compressed and the sizes of the pores filled with air are beginning to decrease while, simultaneously, more physical connections are being made between fibers. Had all pores been eliminated, it would be expected that the value for the resistivity of the material should approach that of amorphous carbon. However, it can never truly reach this value because the pores never fully disappear and also the surfaces of the carbon nanofibers have adsorbed gases that never allow the entangled network of carbon nanofibers to turn into a continuous piece of amorphous carbon. These interfaces cause additional scattering of electrons over what would be expected in amorphous carbon.

## 4. Conclusions

The results of this work indicate that bulk collections of carbon nanofibers can behave as traditional nonwoven materials. This understanding was reached through simultaneous mechanical and electrical analyses. Under tensile load, these nanofibrous nonwovens tend to fail by fiber pullout. During elongation there is no significant enhancement of fiber-to-fiber contact (*i*.*e*., reduction of cross-sectional area) which would be indicated through a reduction in electrical resistance. Under compression, the material behaves elastically, but with significant hysteresis during unloading. Electrical resistance decreases substantially as the material is compressed. The mechanical properties and the reduction in electrical resistance are consistent with the van Wyk model of fiber interaction. The hysteresis during unloading is attributable to small-scale friction and adhesive affects found in fibrous materials. Based on these data, the material is expected to be suitable for applications where cyclic compressive forces are encountered or in applications where an electrical response to deformation may be valuable.
